# A Novel 12q13.2-q13.3 Microdeletion Syndrome With Combined Features of Diamond Blackfan Anemia, Pierre Robin Sequence and Klippel Feil Deformity

**DOI:** 10.3389/fgene.2018.00549

**Published:** 2018-11-19

**Authors:** Domenico Roberti, Renata Conforti, Teresa Giugliano, Barbara Brogna, Immacolata Tartaglione, Maddalena Casale, Giulio Piluso, Silverio Perrotta

**Affiliations:** ^1^Department of Woman, Child and General and Specialized Surgery, University of Campania “L. Vanvitelli” Naples, Italy; ^2^Department of Precision Medicine, University of Campania “L. Vanvitelli”, Naples, Italy

**Keywords:** Diamond Blackfan anemia, Pierre Robin sequence, Klippel feil syndrome, musculoskeletal system development, craniofacial syndromes, craniocervical junction, craniometry

## Abstract

Diamond-Blackfan anemia (DBA) is a rare congenital erythroid aplasia with a highly heterogeneous genetic background; it usually occurs in infancy. Approximately 30–40% of patients have other associated congenital anomalies; in particular, facial anomalies, such as cleft palate, are part of about 10% of the DBA clinical presentations.

Pierre Robin sequence (PRS) is a heterogeneous condition, defined by the presence of the triad of glossoptosis, micrognathia and cleft palate; it occurs in 1/8500 to 1/14,000 births.

Klippel Feil (KF) syndrome is a complex of both osseous and visceral anomalies, characterized mainly by congenital development defects of the cervical spine.

We describe the case of a 22-years-old woman affected by DBA, carrying a *de novo* deletion about 500 Kb-long at 12q13.2-q13.3 that included *RPS26* and, at least, others 25 flanking genes. The patient showed craniofacial anomalies due to PRS and suffered for KF deformities (type II). Computed Tomography study of cranio-cervical junction (CCJ) drew out severe bone malformations and congenital anomalies as atlanto-occipital assimilation (AOA), arcuate foramen and occipito-condylar hyperplasia. Foramen magnum was severely reduced. Atlanto-axial instability (AAI) was linked to atlanto-occipital assimilation, congenital vertebral fusion and occipito-condyle bone hyperplasia. Basilar invagination and platybasia were ruled out on CT and Magnetic Resonance Imaging (MRI) studies. Furthermore, the temporal Bone CT study showed anomalies of external auditory canals, absent mastoid pneumatization, chronic middle ear otitis and abnormal course of the facial nerve bones canal.

The described phenotype might be related to the peculiar deletion affecting the patient, highlighting that genes involved in the in the breakdown of extracellular matrix (*MMP19*), in cell cycle regulation (*CDK2)*, vesicular trafficking (*RAB5B)*, in ribonucleoprotein complexes formation (*ZC3H10*) and muscles function (*MYL6* and *MYL6B*) could be potentially related to bone-developmental disorders. Moreover, it points out that multiple associated ribosomal deficits might play a role in DBA-related phenotypes, considering the simultaneous deletion of three of them in the index case (*RPS26, PA2G4* and *RPL41)*, and it confirms the association among *SLC39A5* functional disruption and severe myopia.

This report highlights the need for a careful genetic evaluation and a detailed phenotype-genotype correlation in each complex malformative syndrome.

## Background

Craniofacial disorders are highly variable developmental anomalies and may occur on their own or with other syndromes (Cielo and Marcus, 2015). Various syndromes, characterized by craniofacial disorders, are also associated with anomalies of the cranio-cervical region (Menezes and Vogel, 2008).

Diamond-Blackfan anemia (DBA) is a congenital erythroid aplasia, that is usually present in infancy as severe hypoplastic macrocytic anemia (Clinton and Gazda, 1993) and it has to be differentiated by diseases with similar onset such as Pearson syndrome (Tumino et al., 2011). It affects about 5 to 7 cases/million live births per year (Lipton et al., 2006). Most cases are sporadic, while approximately 10 to 25% are familial (Campagnoli et al., 2004; Quarello et al., 2012). It is usually associated with morphological abnormalities (Clinton and Gazda, 1993; Campagnoli et al., 2004).

Approximately 30–40% of patients with DBA suffer for congenital anomalies that may involve head, upper limb, heart and genitourinary systems (Vlachos et al., 2008; Boria et al., 2010). The craniofacial anomalies are the most common and characterized, mostly by hypertelorism and broad flat nasal bridge (Vlachos et al., 2008). The hand deformities include triphalangeal thumb and thenar muscle hypoplasia. There may also be weak radial pulse (Ball et al., 1996). Endocrine dysfunctions are common in DBA and, in order of frequency, these include: adrenal insufficiency, hypogonadism, hypothyroidism, growth hormone deficiency/resistance, diabetes mellitus and diabetes insipidus (Lahoti et al., 2016). Many affected children are below average height for their age, and may have delayed puberty (Ball et al., 1996).

Around 60% of patients with DBA have associated germline mutations (50%) or deletions (10%) in 15 ribosomal protein genes and, in rare cases, GATA1 (Choesmel et al., 2007; Boria et al., 2010; Mirabello et al., 2017). There are no clear genotype-phenotype correlations, with exception of patients with mutations in *RPL5* and *RPL11*, which display a high frequency of developmental anomalies, especially cleft palates and triphalangeal thumbs (Gazda et al., 2012). The most common affected gene is *RPS19* (Draptchinskaia et al., 1999; Campagnoli et al., 2004). Heterozygous mutations or deletions of *RPS26* are relatively uncommon (Doherty et al., 2010; Quarello et al., 2012). It has been already described a patient with KFS associated with DBA due to a point mutation in RPS26 identified only by direct sequencing, missing analysis of the surrounding regions (Cmejla et al., 2011).

Pierre Robin sequence (PRS) is a rare etiologically nonspecific complex, defined by the clinical triad of glossoptosis, retro/micrognathia, and cleft or agenesis of the palate (Printzlau and Andersen, 2004; Butow et al., 2009).

Two forms of PRS have been reported in scientific literature: the syndromic PRS (S-PRS) and the non-syndromic PRS (N-PRS). The first one shows a prevalence of about 35%, compared with the 65% of the N-PRS (Printzlau and Andersen, 2004; Butow et al., 2009). The S-PRS, as in this case, can be associated with several different syndromes including Stickler Syndrome, Velo-cardio-Facial Syndrome, Fetal Alcohol Syndrome, mandibular Syndrome and trisomy 18.

Few studies have described the relation between KFS and PRS (Judge et al., 1999; Molnar et al., 2007; Al-Ani et al., 2009; Butow et al., 2009), and we have been able to find only 4 previous cases in literature until now.

Klippel-Feil (KF) syndrome is also a rare disease, that occurs from 1 in 40,000 to 42,000 newborns worldwide, with a slightly higher occurrence in females (Thomsen et al., 1997; Tracy et al., 2004). Symptoms of KFS are cervical vertebra fusion syndrome, KF deformity, and KF sequence disorder, characterized by abnormal fusion of two or more cervical vertebrae, which is present from birth with other osseous anomalies (Tracy et al., 2004; Samartzis et al., 2006; Conforti et al., 2012). The KFS has a heterogeneous clinical presentation and aetiology. Although the majority of cases are un-classified, four different genes have been described as causing this disease: two with an autosomal dominant transmission (*GDF6* – KFS1 and *GDF3* – KFS3) (Tassabehji et al., 2008; Ye et al., 2010) and two with an autosomal recessive one (*MEOX1* – KFS2 and *MYO18B* – KFS4) (Mohamed et al., 2013; Alazami et al., 2015).

## Case presentation

### Subject and clinical features

We describe the case of a 22 year-old woman (II2), known to be affected by PRS. She is the second-born of a mother who had had three term pregnancies (Figure S1). The two brothers are in good health, without signs of congenital abnormalities. The pregnancy was 38 weeks. After a natural childbirth, she weighed 2.1 kg and showed neonatal respiratory distress syndrome. She displayed a typical PRS (micrognathia, glossoptosis, cleft palate) and triphalangeal thumbs. She was also diagnosed with a congenital perimebranous ventricular defect, without haemodynamic effects.

On follow-up examinations, a neurodevelopmental delay was observed: she gained head control at 4–5 months, the ability to sit and to stand unassisted, respectively, at 11 months and at 13 months, and learned to walk, precariously, at 20 months. By this time, she gained a poor verbal language.

In her first months of life, she was hospitalized for the management of her congenital abnormalities; therefore, she was diagnosed with an inherited hyporigenerative anemia that required regular blood transfusion therapy throughout her life. She is currently transfused with four units of packed red blood cells per month.

Genetic characterization of the congenital anemia by multiplex ligation-dependent probe amplification (MLPA) assay led to the discovery of a *de novo* chromosomal deletion involving *RPS26* (data not shown), allowing diagnosis of DBA (Doherty et al., 2010; Quarello et al., 2012).

The clinical examination on admission to our institute, when she was 21 years old, showed a peculiar face, skeletal abnormalities in a complex malformation syndrome and a mental deficiency. Dysmorphic facial features included, beside PRS, prominent nose bridge, low-set ears, bilateral external auditory meatus abnormalities, ocular asymmetry with buphthalmos, intermittent exotropia with left eye dominance, severe myopia, tooth decay and cavities. In addition, skeletal malformations consisted mainly of short stature, right-convex thoracic scoliosis with dorsal hump, hip dysmetria with heterometry of lower limbs, shortness and clinodactyly of fingers with hypoplasia of the distal phalanges of 1th fingers, cutis laxa with characteristic wrinkled palms and soles and multiple skin nevi. She also had several endocrinological alterations as primary amenorrhea, mild hyper-prolactinaemia and moderate familiar hyper-cholesterolaemia.

She showed trigeminal nerve palsy, bilateral mixed hearing loss, rhinolalia, dysarthria and acquired dysphagia for solid foods. She suffers by severe neck pain. Functional limitations in shoulder abduction were detected with lower limbs antigravity muscle weakness and decreased functional ability. Reflexes were normal, except for a bilateral indifferent plantar response.

She was properly informed about all the procedures and about the intent of anonymously publishing the data obtained; to confirm her acceptance, she signed informed consent forms agreeing to the procedures and to publication.

### Radiological features

The complex physical malformations required a neuro-radiological evaluation with Computed tomography (CT) study of the temporal bone, cranio-cervical junction (CCJ) and cervical spine.

Brain and spine Magnetic Resonance Imaging (MRI) studies were performed to evaluate neuro-radiological anomalies.

**Figure 1:**
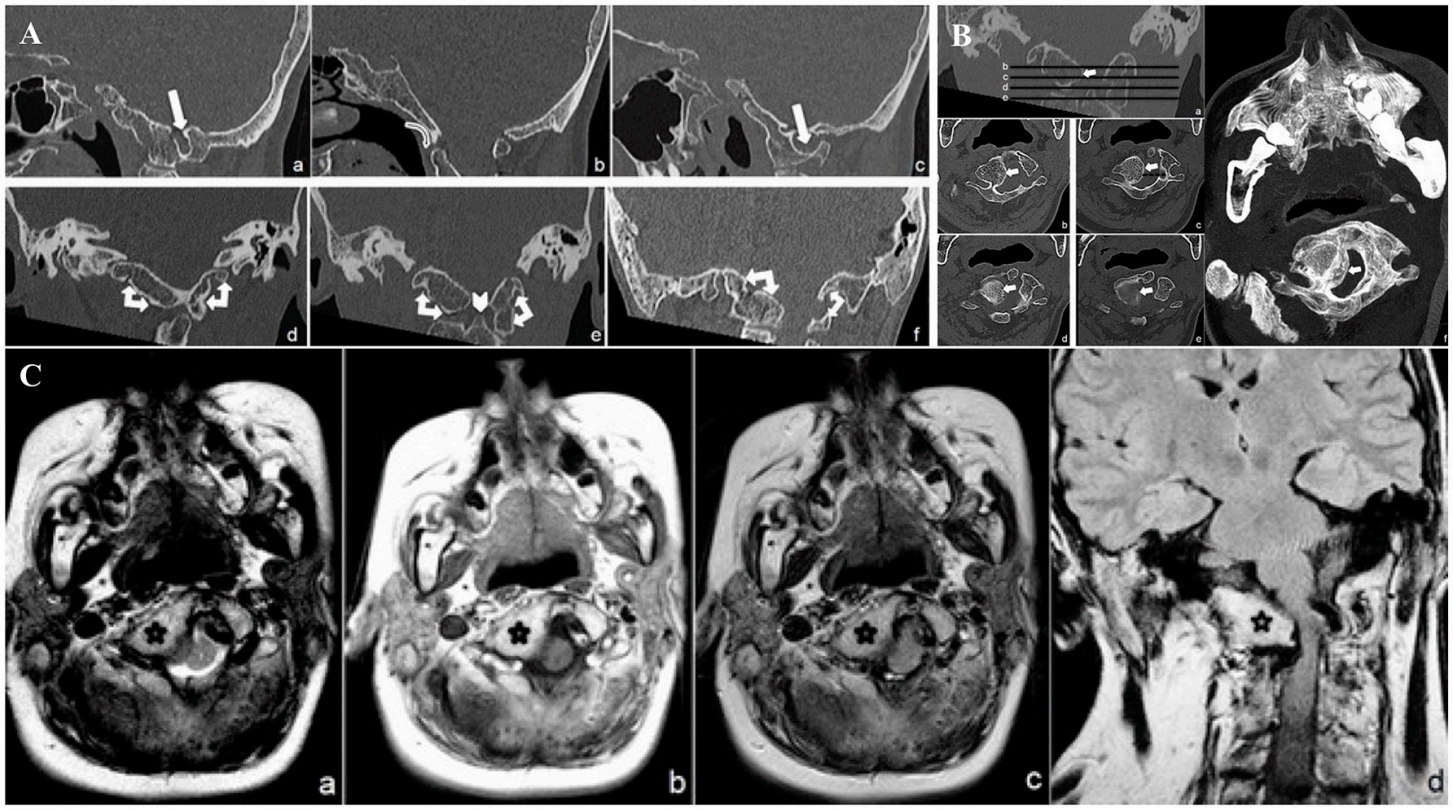
**(A)** CT minimum intensity projection (MIP) Sagittal (a: right parasagittal plane, b: midsagittal plane, c: left parasagittal plane) and Coronal (d: anterior atlanto-occipital complete fusion, e: lateral masses atlanto-occipital bilateral complete fusion, f: posterior atlanto-occipital partial fusion) CCJ reconstruction. Complete C 1 anterior arch and lateral masses fusion and partial fusion of the posterior arch to the occipital foramen (double-headed arrows). Foramen arcuate (white harrows). Note the “comma” appearance of complete anterior atlanto-occipital fusion (curved harrow) and the smaller odontoid process (arrowhead). **(B)** CT Coronal (a) and transverse minimum intensity projection (MIP) (b–e) and transverse shaded surface display (SSD) (f) reconstructions of narrowed foramen magnum due to right occipito-condylar hyperplasia (withe arrows). **(C)** MR transverse T2 (a), T1 (b), FLAIR (c) and coronal FLAIR (d) weighed sequences, show right ventrolateral compression of the lower medulla and upper cervical spinal cord (white arrow) at the level of narrowed foramen magnum due to CCJ complex malformation (black star).

CCJ study focused on bone malformations, showing atlanto-occipital assimilation (AOA) caused by a complete fusion of the C1 anterior arch and lateral masses and by partial fusion of the posterior arch to the foramen magnum. Arcuate foramen was also observed on the left side (Figure 1A). The atlanto-dental interval (AADI: distance, on the sagittal plane, between the anterior assimilated C1 arch and the odontoid process) (Zong et al., 2017) appeared increased (10 mm) suggesting an atlanto-axial instability (AAI) (Figure 1A), defined for atlanto-dental interval >3 mm (Gamble and Rinsky, 1985; Ferreira and Botelho, 2015).

The odontoid process was smaller (Figure 1A) than normal average (Zong et al., 2017). Sites of embryogenesis for the atlas' anterior arch and the atlantoaxial ligaments system are the same, so that the assimilation of the anterior arch of the atlas is often associated with AAI (Ferreira and Botelho, 2015). Very few authors already reported, as in this case, the association between PRS and AAI (Gamble and Rinsky, 1985; Molnar et al., 2007) due to AOA. The patient is affected as well by a right occipito-condylar hyperplasia, producing narrowing and malformation of both the foramen magnum and the upper cervical spine canal (Figure 1B). This hyperplasia causes the compression and displacement of the right side of the bulbo-medullary junction (Figure 1C). Basilar invagination (radiologically defined when the tip of the odontoid is located above the Chamberlin line) (Molnar et al., 2007) and platybasia (flattening of the skull base) were ruled out on both CT and MRI (Kisker et al., 1997; Zong et al., 2017).

**Figure 2:**
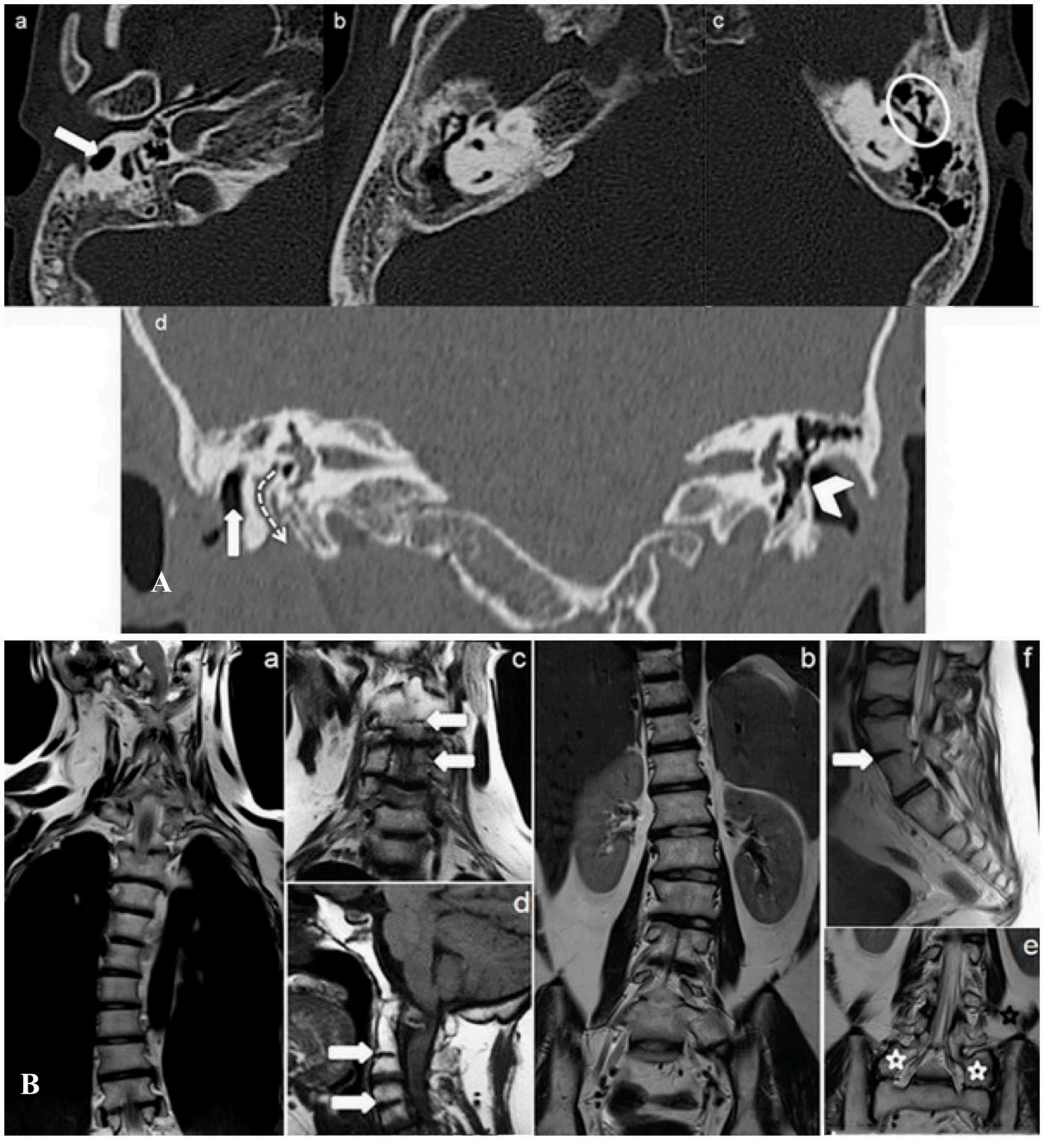
**(A)** Transverse (a–c) and Coronal (d) minimum intensity projection (MIP) CT temporal bone study. Right external auditory canal malformation (a) (white arrow); right chronic otitis media (b) (white star); ankyloses of left ossicular chain (c) (white circle); right widening of facial canal (third segment) (dotted arrow) and partial calcification of left tympanic membrane (arrowhead) (d). **(B)** MR Coronal T2 (a–c, e), Sagittal T1 (d), and Sagittal T2 (f) weighed sequences whole spine study. The study shows, besides rotoscoliosis (a, b), cervical (C 2–C 3, C 4–C 5) (d, f) and lumbar (L 4–L 5) vertebral fusion (white arrows), and L 5 bilateral sacralisation (e) (white stars) (KFS type II malformation).

Temporal bone CT study showed right hypoplasia and anomalous course of the external auditory channels, absence of pneumatization at the right mastoid, a chronic right ear otitis media, widening of the third part of the ipsilateral facial nerve bone canal, partial calcification of the left tympanic membrane and ossicular chain ankyloses (Figure 2A).

Whole spinal cord MRI study added further information, showing not only rotoscoliosis, but also cervical (C2 – C3, C4 – C5) and lumbar (L4 – L5) vertebral fusion and bilateral L5 sacralisation (Figure 2B). The spine malformations led to a diagnosis of KFS type II (Samartzis et al., 2006), without malformations neither of the meninges nor of the nervous system.

### Genetic characterization

**Figure 3:**
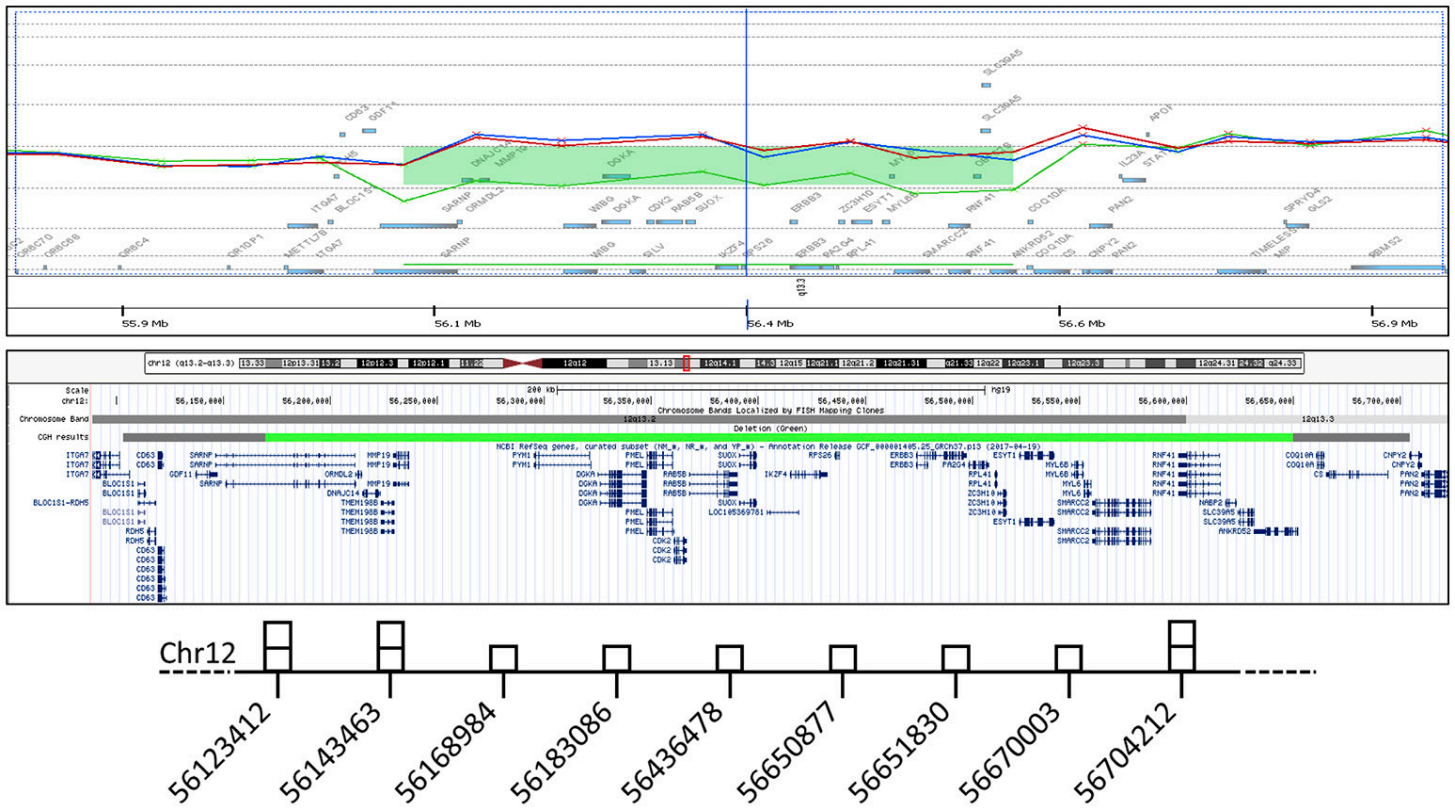
In the gene view (top), the probe distribution and signal intensity are shown for the index patient (green) and her parents (red and blue), with a green bar indicating the deletion detected with 8 probes. In the UCSC graphic view (middle), a similarly colored bar corresponds to the minimal aberration length, while the flanking grey bars indicate the 5' and 3' breakpoint boundaries. The results of genomic quantification by real-time PCR are schematically represented (bottom). The middle base-pair position of each amplicon along chromosome is surmounted by a square corresponding to the number of detected copies.

Dysmorphic features and malformations observed in the patient suggested to further investigate the initially identified genomic deletion affecting *RPS26*. We then carried out array-CGH analysis in the proband and her parents, confirming the presence of a wider *de novo* heterozygous microdeletion on chromosome 12q13.2-q13.3 (Figure 3). By Real-Time PCR, we further refined 5' and 3' breakpoint boundaries of the deletion, resulting in a minimal critical region of about 500 Kb in size (Figure 3 and Table S1). The microdeletion encompassed at least 26 coding genes (Table 1) and expression profiling by Taqman assay confirmed about 50% reduction of the expression for many of these genes (Table 1; data not shown). Some of them, in particular *SARNP, DNAJC14, ORMDL2, DNAJC14, ANKRD52, COQ10A* and *CS*, although heterozygously deleted, were not tested for expression (Table 1). At least three genes (*MMP19, RPS26* and *SLC39A5*) already cause autosomal dominant diseases, while further 12 genes may be sensitive to haploinsufficiency, as they showed a pLI > 0.75 (Table 1). In addition, no similar deletions were annotated in Decipher (decipher.sanger.ac.uk) or have been previously described in literature.

**Table 1:** List of genes deleted in the described patient, with results of Taqman assay and already associated inherited diseases.

**RefSeq ID**	**Gene name**	**Definition**	**pLI**	**Taqman**	**Phenotype**	**MIM**	**Inheritance**
NM_033082	*SARNP*	SAP domain containing ribonucleoprotein	0,99	NT		610049
NM_032364	*DNAJC14*	DnaJ (Hsp40) homolog, subfamily C, member 14	1,00	NT		606092
NM_014182	*ORMDL2*	ORM1-like 2 (S. cerevisiae)	0,15	NT		610074
NM_002429	*MMP19*	Matrix metallopeptidase 19	0,00	↓	Cavitary optic disc anomalies	611543	AD
NM_032345	*PYM1*	PYM homolog 1, exon junction complex associated factor	0.86	↓		
NM_201554	*DGKA*	Diacylglycerol kinase, alpha 80kDa	1,00	↓		125855
NM_006928	*PMEL*	Premelanosome protein	0,00	↓		155550
NM_001798	*CDK2*	Cyclin-dependent kinase 2	0,96	↓		116953
NM_002868	*RAB5B*	RAB5B, member RAS oncogene family	0,22	↓		179514
NM_000456	*SUOX*	Sulfite oxidase, nuclear gene encoding mitochondrial protein	0,00	↓	Sulfite oxidase deficiency	272300	AR
NM_022465	*IKZF4*	IKAROS family zinc finger 4 (Eos)	0,95	↓		606239
NM_001029	*RPS26*	Ribosomal protein S26	0,75	↓	Diamond-Blackfan anemia 10	613309	AD
NM_001982	*ERBB3*	V-erb-b2 erythroblastic leukemia viral oncogene homolog 3 (avian)	0,00	↓	Lethal congenital contractural syndrome 2	607598	AR
NM_006191	*PA2G4*	Proliferation-associated 2G4, 38kDa	1,00	↓		602145
NM_021104	*RPL41*	Ribosomal protein L41	0,04	↓		613315
NM_032786	*ZC3H10*	Zinc finger CCCH-type containing 10	0,79	↓		
NM_015292	*ESYT1*	Extended synaptotagmin-like protein 1	0,00	↓		616670
NM_002475	*MYL6B*	Myosin, light chain 6B, alkali, smooth muscle and non-muscle	0,00	↓		609930
NM_021019	*MYL6*	Myosin, light chain 6, alkali, smooth muscle and non-muscle	0,26	↓		609931
NM_003075	*SMARCC2*	SWI/SNF related, matrix associated, actin dependent regulator of chromatin, subfamily c, member 2	1,00	↓		601734
NM_005785	*RNF41*	Ring finger protein 41	0,86	↓		
NM_024068	*NABP2*	Nucleic acid binding protein 2	0,96	↓		612104
NM_173596	*SLC39A5*	Solute carrier family 39 (metal ion transporter), member 5	0,00	↓	Myopia 24, autosomal dominant	615946	AD
NM_173595	*ANKRD52*	Ankyrin repeat domain 52	1,00	NT		
NM_144576	*COQ10A*	Coenzyme Q10 homolog A (S. cerevisiae), nuclear gene encoding mitochondrial protein	0,12	NT		
NM_004077	*CS*	Citrate synthase, nuclear gene encoding mitochondrial protein	1,00	NT		118950

*Genes are listed as by their locus on chromosome 12; genes already linked to diseases are reported with their associated diseases and inheritance. Taqman assays were used to test the expression levels of the core of this deletion. For each gene, pLI annotated in Exac Browser (http://exac.broadinstitute.org) is reported. A gene with pLI ≥ 0.9 is considered as extremely intolerant to Loss of Function (Lofrese et al., 2015). NT, Not tested; AD, Autosomal dominant; AR, Autosomal Recessive*.

### Discussion

The index patient shows features at the boundaries of the three reported syndromes.

The hypoplastic mandible, the glossoptosis and the U-shaped cleft palate are strictly related to PRS and contribute to airway obstruction (Butow et al., 2009; Cielo and Marcus, 2015) as found in the described patient. In PRS, there is a 10.5% incidence rate of ear malformations (Breugem et al., 2016), which consist of multiple architectural anomalies involving the entire ear, including abnormal auricles, anomalies of the ossicular chain, abnormal stapes footplates and middle ear infection. Middle ear disturbances, as in the index case, are common and are mostly related to the cleft palate and associated to the Eustachian tube dysfunction (Sando et al., 1988; Yamaguchi et al., 1990; Robinson et al., 1993; Gruen et al., 2005). Chronic mucotympanum, chronic middle ear otitis, tympanic membrane retraction pockets and choleastotomas are the most frequent issues related to the middle ear (Gruen et al., 2005). Other anomalies include abnormal course of the facial nerve, as in the described case (Gruen et al., 2005; Rotondo et al., 2010), abnormal insertion of the tensor tympani tendon, ankylosis of the ossicules, and anomalous stapedial footplates, most of which can be related to anomalies of the branchial arches.

Typical elements of KFS are, instead, vertebral fusions, short and webbed neck, decreased range of motion in the cervical spine, deformed chest wall, high placed scapulae, abnormal curvature of the spine (scoliosis), raised scapula (Sprengel's scapula), rib defects, low hair line. The index case shows KF deformity with multiple fusions of non-contiguous cervical and lumbar vertebrae corresponded to type II of KFS, described by Samartzis et al. (Samartzis et al., 2006).

Radiological evaluation pointed out diffuse and severe CCJ bone malformations.

PRS and KFS could both display failure of segmentation between the fourth occipital sclerotome and the first cervical sclerotome resulting in AOA (Menezes and Vogel, 2008; Smoker and Khanna, 2008). The displacement of the assimilated atlas and the right occipito-condylar hyperplasia resulted, instead, in a narrowed foramen magnum, which was responsible for both severe compression of the underlying bulbo-medullary junction and AAI (Gamble and Rinsky, 1985; Yamaguchi et al., 1990; Molnar et al., 2007; Rotondo et al., 2010; Ferreira and Botelho, 2015; Zong et al., 2017). The described Occipito-condylar hyperplasia is an extremely rare congenital entity and only 3 other cases were previously described (Smoker and Khanna, 2008; Rojas et al., 2009; Lofrese et al., 2015). This bone malformation may be due to the excessive growth of the proatlas during the embryogenesis (Smoker and Khanna, 2008; Rojas et al., 2009; Lofrese et al., 2015). Upon birth, the displaced AOA and the overall shift in the head's balance could cause progressive growing of osteophytes, gradually constrain the bulb and the spinal cord. Therefore, neurological decline may occur due to the progressive degenerative ossification (Rojas et al., 2009; Lofrese et al., 2015; Shih et al., 2015). The ossification on the ligamentous interface and ligamentous holding forces may also increase axial instability (Gamble and Rinsky, 1985; Yamaguchi et al., 1990; Molnar et al., 2007; Rotondo et al., 2010; Ferreira and Botelho, 2015; Zong et al., 2017).

The patient's CCJ imbalance (due to skull-cervical spine malformation), coupled with the lack of stable articular joints, exposes her to a high risk of sudden neurological manifestations. It has been shown that common pathways exist between the neural cresta and the paraxial mesoderm embryogenesis during CCJ development (Menezes and Vogel, 2008).

Among genes deleted in the index case, three of them are involved in ribosomal biogenesis (*RPS26, PA2G4, RPL41*). Ribosomes catalyze for protein synthesis and their function impairment have been extensively involved in DBA pathogenesis. The deletion of this gene cluster could aggravate hematological features linked to DBA. Other genes in the deleted region are related to different pathways.

*MMP19* is a gene related to autosomal dominant inherited congenital cavitary optic disc anomalies (Moore et al., 2000), encoding for a zinc-binding endopeptidases that degrades several components of the extracellular matrix. Zinc is an essential cofactor for hundreds of enzymes, thus being fundamental for hundreds of functions.

The identified deletion also included *SLC39A5* that encodes for a protein showing structural characteristics of zinc transporters and it has been linked with an autosomal dominant severe form of myopia (Guo et al., 2014), as in the described patient.

Other genes involved in the deletion where expected to be extremely intolerant to Loss of Function (LoF) because of their pLI.

SAP domain containing ribonucleoprotein (*SARNP*) has a putative role in cell cycle progression acting as a single-stranded DNA binding protein (Fukuda et al., 2002).

*DNAJC14* (DnaJ Heat Shock Protein Family (Hsp40) Member C14) regulates target proteins' export from the endoplasmic reticulum to the cell surface and it seems to be involved in protection from flavivirus infections (Yi et al., 2011). The exon junction complex (EJC) serves as a positional landmark for the intron exon structure of genes; the index patient has a deletion of *PYM1*, a key member of this complex, actin as a disassembly factor; it also associates with the 40S ribosomal subunit (Gehring et al., 2009). *DGKA*, instead, encodes for a Diacylglycerol kinase, ultimately removing it; it plays a role in intracellular signaling and phospholipid synthesis. Cell cycle control has been vaguely related to developmental syndromes (Tessadori et al., 2017). The index patient has an allelic deletion for *CDK2*, a key cell cycle related serine/threonine protein kinase. *IKZF4* encodes a zinc-finger transcription factor required during early B cell development. The protein encoded by *SMARCC2* displays helicase and ATPase activities, whose enzymatic disruption often leads to development disorders. Type 1 cytokine receptor signalling impairment has been, instead, linked previously to congenital Immunodeficiency, although their functions are redundant; *NABP2* encodes for a Single-stranded DNA-binding protein, usually necessary for several DNA-related metabolic processes.

Ankyrin repeat domain 52 (*ANKRD52)* is a protein acting as a regulatory subunit of protein phosphatase 6 (PP6), involved in the recognition of phosphoprotein substrates (Watanabe et al., 2018). Citrate synthase is, instead, a protein codified by *CS*, acting as a Krebs tricarboxylic acid cycle enzyme catalazying the synthesis of citrate from oxaloacetate and acetyl coenzyme A (Hayward and Berendsen, 1998).

New studies focused on the role played by these genes, in developmental control and their regulatory functions and accurate craniometrical studies of CVJ anomalies, may offer early diagnosis, new therapeutic targets and specific treatment before neurological damage occurs.

This case-report points out that DBA, and mostly PRS and KF have a further genetic heterogeneity and it underlines the need for a careful genetic evaluation and a detailed phenotype-genotype correlation in each complex malformative syndrome.

## Data availability

Data sharing is not applicable to this article as no datasets were generated or analyzed during the current study.

## Ethics statement

We confirm that any aspect of the work covered in this manuscript that has involved the human patient has been approved by the ethical committee of our institution and has been conduct according to the The Code of Ethics of the World Medical Association (Declaration of Helsinki) for experiments involving humans and to general Good clinical practice principles and respecting clinical expertise, patient values, and the best research evidence for the patient's care. The patient and her mother signed informed consents to undergo the procedures described. Moreover, a written informed consent has been obtained for publication in print and electronic form from the patient and her mother.

## Author contributions

DR takes care of the patient, performed the Taqman assays, coordinated the study evaluation and wrote the manuscript. RC performed the radiological evaluation and wrote the manuscript. TG performed the genetic assays. BB performed the analysis of the radiological data. IT and MC take care of the patient and performed the clinical evaluation. GP supervised the genetic analysis and wrote the manuscript. SP supervised the analysis and the manuscript preparation.

### Consent for publication

A written informed consent has been obtained for publication in print and electronic form from the patient and her mother.

### Conflict of interest statement

The authors declare that the research was conducted in the absence of any commercial or financial relationships that could be construed as a potential conflict of interest.

## Abbreviations

AADI: Anterior atlanto-dental interval
AAI: Atlanto-axial instability
AOA: Atlanto-occipital assimilation
CCJ: Cranio-cervical junction
Cgh-Array: Comparative genomic hybridization Array
CT: Computed tomography
DBA: Diamond-Blackfan anemia
KF: Klippel Feil
MRI: Magnetic resonance imaging
PRS: Pierre Robin sequence.
