# Supplementary material for: A Novel 12q13.2-q13.3 Microdeletion Syndrome With Combined Features of Diamond Blackfan Anemia, Pierre Robin Sequence and Klippel Feil Deformity
Source: Front Genet. 2018 Nov 19;9:549. doi: 10.3389/fgene.2018.00549 (PMC6262175; doi:10.3389/fgene.2018.00549)
Supplement: Supplementary file 1 [file Table_1.docx]

**Supplementary Information**

**A novel** **12q13.2-q13.3 microdeletion syndrome with combined features of Diamond Blackfan Anemia, Pierre Robin sequence and Klippel Feil deformity.**

*Roberti* Domenico^1#^, MD-PhD, *Conforti* Renata^2#^, MD, *Giugliano* Teresa^2^, PhD, *Brogna* Barbara^2^, MD, *Tartaglione* Immacolata^1^, MD, *Casale* Maddalena^1^, MD-PhD, *Piluso* Giulio^2^, MD-PhD, *Perrotta* Silverio^1*^, MD.

1. Department of Woman, Child and General and Specialized Surgery - University of Campania “L. Vanvitelli”, Naples, Italy.

2. Department of Precision Medicine, University of Campania “L. Vanvitelli”, Naples, Italy.

# These authors contributed equally to this work.

*Corresponding Author: Prof. Silverio Perrotta, [Silverio.Perrotta@unicampania.it](mailto:Renata.Conforti@unicampania.it),

phone: +0815665421, fax: +390815665690

**Materials and Methods**

*Multiplex ligation-dependent probe amplification (MLPA)*

MLPA analysis was performed using the SALSA MLPA P212 DBA kit (MRC-Holland, Amsterdam, The Netherlands), according to the manufacturer’s recommendations.

Briefly, denatured genomic DNA (100 ng) was added to the MLPA mix and probes were allowed to anneal overnight before the subsequent ligation reaction was performed. PCR was carried out with 6-carboxyfluorescein (FAM)-labeled primers using 5 μL of the ligation reaction as the template. The PCR products were then separated on an ABI 3130xL automatic DNA sequencer (Life Technologies), including at least three normal DNA samples in each batch of the MLPA assays for the next normalization of results.

MLPA data analysis was performed using the Coffalyser v9.4 package (MRC-Holland, Amsterdam, The Netherlands). Relative amounts of probe-amplified products were compared with reference samples to determine the copy number of the target sequences. Values under a threshold of 0.7 and over a threshold of 1.3 for multiple adjacent probes, indicate the presence of a deletion or duplication respectively.

*Radiological examination*

Cardiac ultrasound study confirmed the inter-ventricular septal defect. A chest x-ray (XR), performed before surgery for dental remediation, showed a right raised scapula and fusion of the middle arches of the right third and fourth ribs. The complex physical malformations required also a neuro-radiological evaluation with CT study of the temporal bone, cranio-cervical junction (CCJ) and cervical spine.

Moreover, brain and spine MRI studies were performed to exclude any brain and spinal cord neuro-radiological anomalies. Throughout MRI brain study, we also focused on the sellar region because of endocrinological dysfunctions.

*CT data acquisitions*

The temporal bone High Resolution Helical Multi–detector CT study (HRCT) is supported by basicranium and CCJ study (Siemens Medical Systems) (row 4 slices acquisition on the transverse plane, 120 kV, 180 mAs, 1 second rotation time, 0.5 mm thickness, 0.5 mm collimation, 0.2 reconstruction increment, 1 mm table feed and rotation, matrix: 512x512, 9 cm field of view, multiplane reformatted images 1mm section thickness and 0.5 overlap mm). CT scan with 3D reconstruction was acquired to check the bone dimorphisms of the patient

*CCJ craniometric analysis*

The images were assessed on the radiological workstation center, and all measurements were performed using the picture archiving communication system (PACS) by 2 researchers. CCJ study was focused on bone deformities and on craniometric evaluation, supported by craniometrics measures on CT imaging. Our purpose was to rule out bone malformations and possible atlanto-axial instability (AAI), basilar invagination (BI) and platybasia. The measures drawn out were the following. Antero-posterior (sagittal) length (FM-L) and horizontal width (FM-W) of the foramen magnum (FM) which were both measured respectively on the transverse plane as the greatest distance between the anterior and posterior rims of the FM and the greatest distance between its left and right lateral surface.

TP [The anterior atlanto-dental interval (AADI) is the distance, on the sagittal plane, between the anterior assimilated C1 arch and the odontoid process. AAI is defined for AADI>3mm.]

Accordingly the lateral atlando-dental-interval (LADI), as distance between the odontoid process and lateral mass of the atlas, was measured bilaterally on coronal plane.

The posterior atlanto-dental interval (PADI) is the distance between the odontoid process and the posterior assimilated C1 arch. The level of spinal canal encroachment diagnosis has been defined for PADI<13mm.

Both sagittal inferior C1 right and left facet angle (SIA) and coronal inferior C1 right and left facet angle (CIA) are measured crossing the C1 lateral mass; they were formed as a line extending from the facet to a horizontal line. The SIA, were measured on right and left parasagittal plane. The right and left CIA were measured on coronal plane.

The height of odontoid process (HO) of C 2, defined as the distance between the apices and the superior articular facet of the C2 vertebra, was measured on coronal plane.

We have evaluated the possible BI and Platybasia on both CT and MRI studies on sagittal plane. Therefore, we have drawn the distance between the apices of odontoid process and the Chamberlin line (CHLD). Chamberlin line (CHL) was drawn on the sagittal plane from the posterior edge of the hard palate to the opisthion; from here, a perpendicular line was then drawn to the apices of odontoid process of C 2. The odontoid projecting above this line> 5mm was diagnostic as Basilar Invagination.

The basal angle (BA) was measured as the angle formed by an intersection between a line from the nasion to the dorsum sellae and a line from the dorsum sellae to the basion. The diagnosis of platybasia (flattening of the skull base) is evident when the BA is >133°[14, 19, 21].

The clivus-canal angle (CCA) is an angle formed by a line from the dorsum sellae to the basion and a line extending from the dorsal portion of C 2 body. Ventral spinal cord compression may occur with angles less than 150°.

*MRI protocols*

MRI study was performed with a 1.5 Tesla (1.5 MRI, Siemens Medical System). The images obtained included the following:

- Constructive interference in steady state (CISS) sequence (TR: 5.40 TE: 2.30, slice thickness: 1.0 and flip angle: 65 degrees), for inner ears study.

- Brain MRI transverse FLAIR weighted sequence (TR:8890, TE:123 slice thickness: 5.0), transverse T2 weighted sequence (TR:3350, TE:116, slice thickness:5mm), transverse diffusion-weighted weighted images (DWI) (TR:4800 TE:106, slice thickness:5mm), three-dimensional (3D) multiplanar gradient recalled (MPGR) weighted sequences (TR:487, TE:9.70, Slice thickness 4mm)

- Coronal pre-contrast T1-weighted spin echo (SE) scans were obtained for the study of the sellar region using the following protocol: repetition time/echo time, 549/8.7 msec; 192 × 256 matrix; two excitations; 12-cm field of view; and interleaved sections, 3 mm in thickness without intersection gap. Scan time was 5.10 min. The study was repeated after the per venam administration of gadolinium contrast material (0.01 mmol/kg gadopentetate dimeglumine)

- Whole Spine T2 and T1 weighted sequence (TR:3320, TE: 97, slice thickness: 3mm) on sagittal, transverse and coronal plane.

*Array CGH analysis*

Genomic DNA was analyzed using the SurePrint G3 CGH ISCA v2 8X60K format (Agilent Technologies, USA). Labeling and hybridization were performed according to the manufacturer’s specifications (Agilent Oligonucleotide Array-Based CGH for Genomic DNA Analysis protocol, version 6.1; Agilent Technologies, USA). Scanned array image was analyzed using Feature Extraction software (version 10.5.1.1; Agilent Technologies, USA). Graphical overview and analysis of data were obtained using DNA Analytics as part of Agilent Genomic Workbench software (version 7.0.4.0; Agilent Technologies, USA).

To identify duplications and deletions we used the standard set-up of ADM-2 algorithm for data that passed QC metrics testing. The threshold applied to the algorithm was set to 6. An aberration filter was set to select aberrant regions with at least three targets showing the same direction in copy-number change, and to exclude aberrant regions if the average log2 ratio within the region was less than the value of DLRSpread.

Identified CNVs were compared with the Database of Genomic Variants (http://dgv.tcag.ca/) and with the Decipher database (https://decipher.sanger.ac.uk/) to facilitate interpretation.

*CNV validation*

CNV validation was performed by quantitative amplification of specific genomic regions (primer pairs available in supplementary Table S1) on CFX96 Real-Time PCR Detection System (Bio-Rad Laboratories, USA) using iQ SYBR Green Supermix (Bio-Rad Laboratories, USA), according to manufacturer's instructions. Each assay was performed in triplicate and results were normalized and analyzed using CFX Manager Software Version 1.5 (Bio-Rad Laboratories, USA).

*Taqman Assay*

Gene expressions profiles were analysed by optimized TaqMan quantitative RT PCRs on cDNA produced by the patient’s leukocyte and from a healthy control. Genes’ analysed list is reported; RT-PCRs were performed using TaqMan™ Fast Advanced Master Mix (Applied Biosystems™) according to manufacturer’s instructions. Each reaction was performed using a pair of unlabeled PCR primers and a TaqMan probe with an Applied Biosystems FAM or VIC dye label on the 5’ end and minor groove binder (MGB) and nonfluorescent quencher (NFQ) on the 3’ end; list of the primers is available upon request.

**Supplementary Figure S1 –** *Family Pedigree* (member II2, identified by the arrow, is the proband)

**
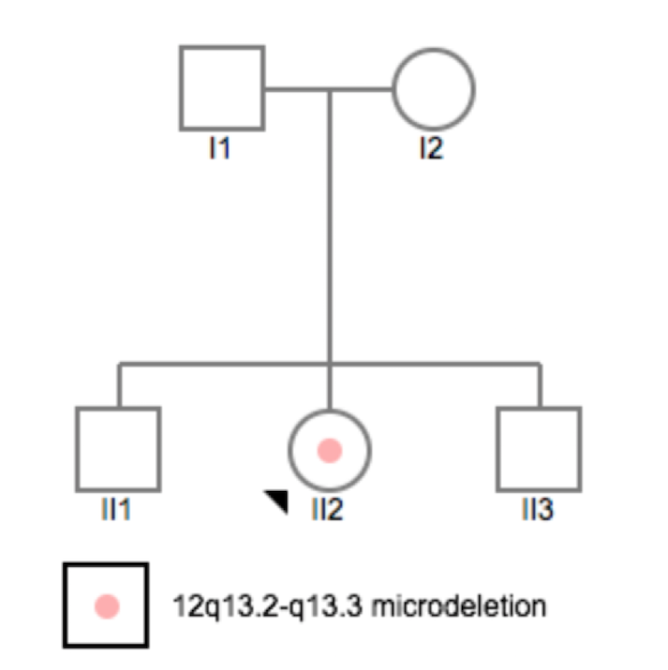
**

**Supplementary Table S1 –** *Primer pairs for CNV validation by qPCR and genomic coordinate of each amplicon.*

| **Forward primer** | **Reverse primer** | **Coordinates on Chr 12 (Hg19)** |
| --- | --- | --- |
| CAATGTGGTCTGTGAGGTTTCCC | GGCCAGAAATCCCCACTCTAGAA | 56123192-56123412 |
| GGGAAAGGAACAGGGAAGAGGAA | AGTAGTTGGCCTTGTAGCGCTTA | 56143216-56143463 |
| AGGTTTAAGCCATTCTCCTGCCT | AAATAGAAATGTGTGGCTGGGCG | 56168764-56168984 |
| ACTTCCATCGAGCCTACATTACCT | TGGACTGGATGGTTGCTGCTTAG | 56182837-56183086 |
| CAGTTTTCCCATCCTGTCGCAG | CTCCAAAGAACAGAGCCTCCAAA | 56436187-56436478 |
| AGTTCTAGGGGTAGGAGGAAGGG | CCCCACAGGTGCTAATGTCAATG | 56650642-56650877 |
| TCCCAACTCACCAGCACATTGAT | CTCCAGCCAGAATCCCTAGAGTG | 56651601-56651830 |
| AATTTCCCACCAATTGAGGCACC | TCCATCTTGCTCCTCTTCTGTCA | 56669727-56670003 |
| TATGTTCCCCTGCTTCCTCATGG | ACTGTGTTGTCTTTTCATGCCTGG | 56703939-56704212 |
